# Unusual Relapse of Primary Central Nervous System Lymphoma at Site of Lumbar Puncture

**DOI:** 10.1155/2014/161952

**Published:** 2014-06-29

**Authors:** Zartaj Ahmed, Ramesh K. Ramanathan, Sunil Ram, James Newell, Maqbool Halepota

**Affiliations:** ^1^Dow Medical College, Dow University of Health Sciences, Baba-e-Urdu Road, Karachi 74200, Pakistan; ^2^Virginia G Piper Cancer Centre, 10460 N. 92nd Street, Suite No. 200, Scottsdale, AZ 85258, USA; ^3^Scottsdale Medical Imaging, 3501 N. Scottsdale Road No. 130, Scottsdale, AZ 85251, USA; ^4^Scottsdale Pathology Associates, 9003 E. Shea Boulevard, Scottsdale, AZ 85260, USA; ^5^Palo Verde Cancer Centre, 10460 N. 92nd Street, Suite 402, Scottsdale, AZ 85258, USA

## Abstract

Primary CNS lymphoma (PCNSL) is a rare non-Hodgkin's lymphoma confined to the CNS. Local relapse of this disease is common, but extracranial or subcutaneous metastasis is rare with only a few cases being reported in literature. We report a 63-year-old male patient, who responded well to treatment for PCNSL but relapsed two and half years later with a lumbosacral nodule at the site of a previous lumbar puncture due to microscopic tumor seeding. Clinicians treating patients with PCNSL must remain alert to the possibility of extracranial solitary relapse even after the resolution of initial disease because prompt treatment can result in a good outcome.

## 1. Introduction

Primary CNS lymphoma (PCNSL) is an aggressive uncommon form of extranodal non-Hodgkin's lymphoma confined to the CNS, but its incidence has increased in both immunocompromised and immunocompetent hosts. Around 1900 new cases are diagnosed in the USA each year. It affects the brain parenchyma, intraocular compartment, cranial nerves, and rarely the spinal cord [1]. It usually presents as a solitary mass and accounts for 3% of all primary brain tumors with a median age of onset of 52 years and a male to female ratio of 1.2–1.7 : 1 [1].

Stereotactic biopsy is the diagnostic procedure of choice revealing CD20^+^ diffuse large B cell lymphoma [2]. Advantage of radiation and chemotherapy over radiation alone is suggested [3]. National Comprehensive Cancer Network (NCCN) guidelines therefore state high dose methotrexate therapy and whole brain radiotherapy as standard treatment [4]. 10–15% of patients are primarily refractory to treatment [5].

Data on the relapse of PCNSL is limited and prognosis is poor, with a 2–4-month survival [6] Relapse is seen in 35–60% of patients after 2 years from initial diagnosis [7] and in 4% of patients after ≥5 years [8]. Most patients relapse in intracranial sites and less than 5% patients relapse in extracranial sites [9]. Isolated systemic relapse without CNS involvement is rare, so much so that Kim et al. claim [5] regular systemic evaluation of extracranial sites may not always be necessary. Hochberg and Batchelor [10] state that systemic dissemination occurs in 7–10% of patients with advanced PCNSL and tends to involve extranodal organs, for example, the kidneys, skin, and testicles. The subarachnoid space is a common site of relapse with subsequent development of leptomeningeal disease. The eye is a potential site of relapse; however, very few relapses occur in the spinal cord.

In patients with late relapses ≥5 years, intracranial sites remained most frequent with only one systemic relapse occurring in the kidney among 230 patients, as reported by Nayak et al. [11].

We report a patient with well documented PCNSL, who relapsed in a site of previous lumbar puncture, without CNS involvement.

## 2. Case Presentation

**Figure 1:**
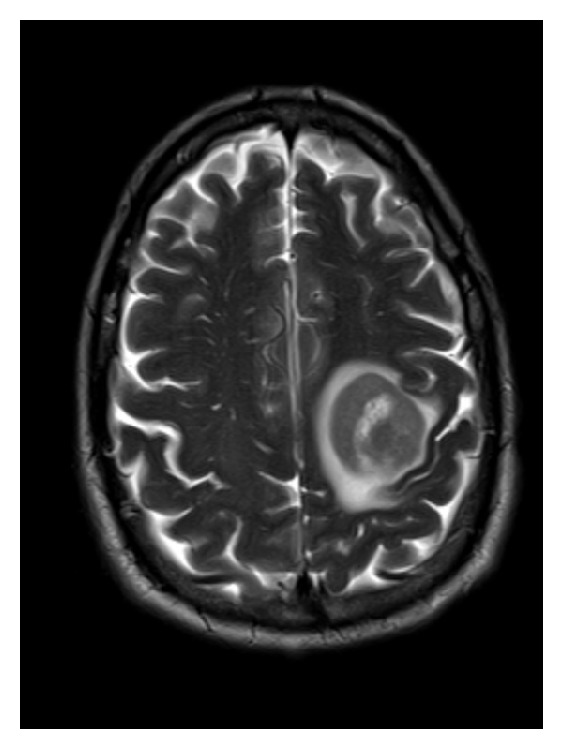
MRI of the brain showed a large mass in the left frontal area T2/FLAIR. Mild surrounding vasogenic edema and mass effect.

**Figure 2:**
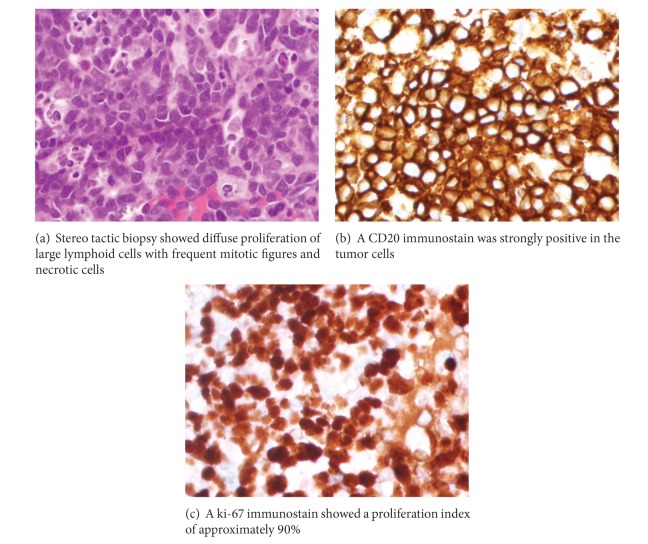
Stereotactic biopsy diagnostic of diffuse large B cell lymphoma.

A 63-year-old man presented, in October 2009, with complaints of right sided hemiparesis. An MRI of the brain showed a large mass in the left frontal area (Figure 1) and a stereotactic biopsy showed that the tumor consisted of a diffuse proliferation of large lymphoid cells with frequent mitotic figures and necrotic cells (Figure 2(a)). A CD20 immunostain was strongly positive in the tumor cells (Figure 2(b)). A ki-67 immunostain showed a proliferation index of approximately 90% (Figure 2(c)). A bcl-2 immunostain was positive whereas immunostains for bcl-1, CD5, and CD10 were negative (not shown). A FISH study was negative for MYC translocation. The overall pathologic findings were diagnostic of a diffuse large B cell lymphoma. Workup for systemic disease including bone marrow biopsy, CT scans, and a lumbar puncture for CSF examination was all negative; therefore, he was diagnosed with primary CNS lymphoma (PCNSL). The workup for HIV disease was negative. The patient was treated with 8 cycles of high dose methotrexate, with significant improvement in his symptoms. He was given adjuvant radiation after completion of his chemotherapy and, in February 2010, acquired complete remission. A posttreatment MRI brain showed no recurrent neoplasm.

He stayed in remission until August 2012, when he presented with a palpable 1.5 cm nodule, in the lumbosacral region, the site of a previous lumbar puncture. A biopsy of the lesion showed a diffuse large B cell lymphoma, consistent with recurrence of the original CNS lymphoma. He underwent local excision followed by chemotherapy, in the form of 4 cycles of the R-CHOP (Rituxan, Cyclophosphamide, Doxorubicin, Vincristine, and Prednisone) regimen, to treat the recurrent nature of his disease. Postchemotherapy consolidation radiotherapy of 3000 Gy in 250 cGy was directed at the lumbosacral spine region and the patient acquired complete remission, with the area showing no palpable mass, hypopigmentation, or wound dehiscence. At the last visit (on 17th of September 2013), the patient continued to be in remission.

## 3. Discussion

Extracranial skin or subcutaneous recurrence of PCNSL is rare with few reported cases in the literature. Al Bahrani et al. [12] reported a case of recurrence in the form of a painless subcutaneous nodule in the leg, a few months after resolution of PCNSL, treated with radiation therapy. Koga et al. [13] reported a patient who two years after treatment of PCNSL presented with a slow growing, erythematous nodule in the forearm. In another case study presented by Ko et al. [14], the patient presented with tumor growth in the right neck and periumbilical areas due to seeding of the PCNSL along a ventriculoperitoneal shunt.

Our patient, who presented with PCNSL and responded well to treatment with high dose methotrexate and radiation, remained in remission for almost two and a half years. He then presented with an extracranial relapse; however, according to the literature, PCNSL only rarely relapses or metastasizes to extracranial regions. The 1.5 cm solitary subcutaneous nodule discovered in our patient at the site of lumbar puncture responded well to treatment with the monoclonal antibody Rituximab and CHOP, which represent the backbone for treating patients with DLBCL [15]. After radiation therapy the patient is in complete remission.

In our review of the existing literature, a previous case on relapse of PCNSL in the lumbosacral region, especially in a site of previous lumbar puncture, could not be found. We speculate that the reason for development of the rare lesion at the site of the lumbar puncture was due to microscopic tumor seeding.

## 4. Conclusion

Clinicians treating patients with PCNSL must remain alert to the possibility of extracranial solitary relapse even after the resolution of initial disease because prompt treatment can result in a good outcome.
